# Atypical Pestivirus and Severe Respiratory Disease in Calves, Europe

**DOI:** 10.3201/eid1708.101447

**Published:** 2011-08

**Authors:** Nicola Decaro, Maria Stella Lucente, Viviana Mari, Francesco Cirone, Paolo Cordioli, Michele Camero, Rossana Sciarretta, Michele Losurdo, Eleonora Lorusso, Canio Buonavoglia

**Affiliations:** Author affiliations: Faculty of Veterinary Medicine of Bari, Valenzano, Bari, Italy (N. Decaro, M.S. Lucente, V. Mari, F. Cirone, M. Camero, R. Sciarretta, M. Losurdo, E. Lorusso, C. Buonavoglia);; Istituto Zooprofilattico Sperimentale di Lombardia ed Emilia Romagna, Brescia, Italy (P. Cordioli)

**Keywords:** HoBi-like pestivirus, respiratory disease, Europe, genetic analysis, biological analysis, viruses, cattle, dispatch

## Abstract

In 2010, a HoBi-like pestivirus was isolated from clinically affected calves in Italy. This European virus reproduced a milder form of disease under experimental conditions and was genetically related to previously reported HoBi-like strains. Isolation of this novel virus from a clinical outbreak may have implications for cattle health and prophylactic programs.

Genus *Pestivirus* (family *Flaviviridae*) includes *Bovine viral diarrhea virus type 1* (BVDV-1) and *Bovine viral diarrhea virus type 2* (BVDV-2), *Classical swine fever virus* (CSFV), *Border disease virus* (BDV), and other pestivirus species detected in wild ruminants (*1**,**2*). In 2004, an atypical pestivirus was isolated from a contaminated batch of calf serum originating from Brazil. This virus, named D32/00_HoBi, was proposed as prototype of a new pestivirus species, BVDV-3 (*3*). Although additional HoBi-like strains have been detected in South America (*4*), currently, there is a unique report of natural infection in cattle caused by a HoBi-like strain, Th/04_KhonKaen, which was isolated from a bovine serum sample collected during an epidemiologic survey for BVDV in Thailand (*5*). However, the virus was not associated with any evident clinical signs. Here we report the biologic and genetic characterization of a HoBi-like strain from Europe that was isolated from cattle during an outbreak of respiratory disease in Italy.

## The Study

A severe outbreak of respiratory disease occurred in a cattle herd in Calabria region, southern Italy, during December 2009–February 2010. The animals were not screened for pestiviruses before their introduction into the herd but were vaccinated regularly for BVDV. Clinical signs appeared in 26 calves, 6–7 months old, and consisted of fever (39.4°–40.1°C), cough, accelerated pulse and breath, seromucoid nasal discharge, and leukopenia. Most animals recovered progressively within 2 weeks after administration of supportive therapy. Two calves died, and necropsy indicated severe tracheitis and bronchopneumonia involving the apical lung lobes.

The nasal discharge of 6 ill calves and the apical lobes of the dead calves were subjected to traditional or molecular assays to detect the main respiratory pathogens of cattle (*6*). All samples tested positive by a TaqMan assay specific for atypical pestiviruses (*7*) and contained RNA copy numbers ranging from 2.57 × 10^3^ to 5.48 × 10^5^ copies/μL of template. The pestivirus strains (Italy-1/10-1 and Italy-1/10-2) detected in the lung samples of 2 calves were successfully isolated on MDBK cells as shown by the positive results of an immunofluorescence assay by using an anti-NS3 monoclonal antibody pool (3A3, 3H4, IF2). Other viral pathogens of cattle, as well as bacteria and parasites, were not detected in the examined samples, with the exception of the lungs of the dead calves, from which *Streptococcus bovis* and *Vibrio* spp. were isolated.

The near full-length genome (12,104 nt) of strain Italy-1/10–1, representative of the pestiviruses circulating in the herd, was determined through PCR amplifications and subsequent sequencing of overlapping fragments (*8*). The nucleotide sequence (GenBank accession no. HQ231763) obtained was analyzed by using NCBI (www.ncbi.nlm.nih.gov) and EMBL (www.ebi.ac.uk) tools. By sequence comparison with reference sequences, strain Italy-1/10–1 had the same genomic organization of other members of the genus *Pestivirus*, consisting of a unique open reading frame of 11,700 nt flanked by 2 untranslated regions (UTRs). By sequence analysis of the near full-length genome, strain Italy-1/10–1 displayed the closest relatedness to atypical pestivirus Th/04_KhonKaen, whereas the nucleotide identities to BVDV-1 and BVDV-2 reference strains were much lower (Table). Percentage identities were similar between the HoBi-like strain from Italy and BDV and CSFV reference isolates. When the informative genomic regions were analyzed, strain Italy-1/10-1 displayed the closest relatedness to strains D32/00_HoBi and CH-KaHo/cont that had been detected in South America.

**Table:** Nucleotide identity of HoBi-like pestivirus strain Italy-1/10-1 with reference pestiviruses in different genomic regions*

Pestivirus species and strain	GenBank accession no.	Nucleotide identity, %			
		Full-length genome	E2	5′ UTR	N^pro^
HoBi-like pestivirus					
Th/04_KhonKaen	FJ040215	90.0	87.7	92.3	89.4
D32/00_HoBi	AY604725 (E2); AY489116 (5′ UTR); AY735486 (N^pro^)	NA	94.2	98.3	95.2
CH-KaHo/cont	EU385605 (E2); AY895011 (N^pro^)	NA	93.4	NA	95.2
Border disease virus					
H2121 (Chamois-1)	GU270877	66.7	58.1	61.8	64.6
Gifhorn	GQ902940	66.7	58.2	65.5	64.2
X818	NC_003679	66.9	59.3	60.7	66.2
Reindeer	AF144618	66.3	58.6	65.0	63.2
Classical swine fever virus					
Brescia X	AY578687	66.9	58.5	66.6	66.6
HCLV	AF531433	66.5	59.0	65.5	65.4
Brescia	AF091661	66.8	59.0	66.1	65.4
Alfort-A19	U90951	66.8	59.0	66.1	65.8
Shimen/HVRI	AY775178	66.8	59.2	66.6	67.0
Riems	AY259122	66.4	58.7	65.5	65.2
Pestivirus of giraffe, H138	AF144617	65.2	58.0	72.8	64.2
Bovine viral diarrhea virus type 1					
ILLNC	U86600	66.3	60.9	69.8	64.0
ZM-95	AF526381	66.2	62.1	65.7	63.6
Oregon-C24V	AF091605	66.4	61.1	68.8	66.2
CP7-5A	AF220247	66.4	62.1	68.2	66.0
SD1	M96751	66.3	60.1	68.8	66.2
Singer_Arg	DQ088995	66.5	61.4	69.3	65.0
KE9	EF101530	67.0	61.4	70.4	65.8
NADL	M31182	65.3	62.0	69.3	66.2
VEDEVAC	AJ585412	66.4	61.3	70.9	66.2
Bovine viral diarrhea virus type 2					
JZ05-1	GQ888686	67.4	59.5	75.0	63.8
New York’93	AF502399	67.1	58.7	74.4	65.0
XJ-04	FJ527854	67.1	59.3	74.4	64.2
C413	NC_002032	67.1	58.5	74.4	63.8
Hokudai Lab/09	AB567658	67.2	59.3	72.8	65.2

*UTR, untranslated region; NA, sequence not available.

Phylogeny was inferred from the full-length genome by using the neighbor-joining method of MEGA4.1 software (*9*). The analyzed pestiviruses clustered into 6 monophyletic clades, with strain Italy-1/10–01 forming a unique cluster with strain Th/04_KhonKaen, which was clearly separated from the other pestivirus species (Figure, panel A). A similar cluster was observed in the trees constructed on the E2, 5′ UTR and N^pro^ sequences, where the HoBi-like strain from Europe formed a tight subcluster with South America viruses (Figure, panels B–D).

**Figure:**
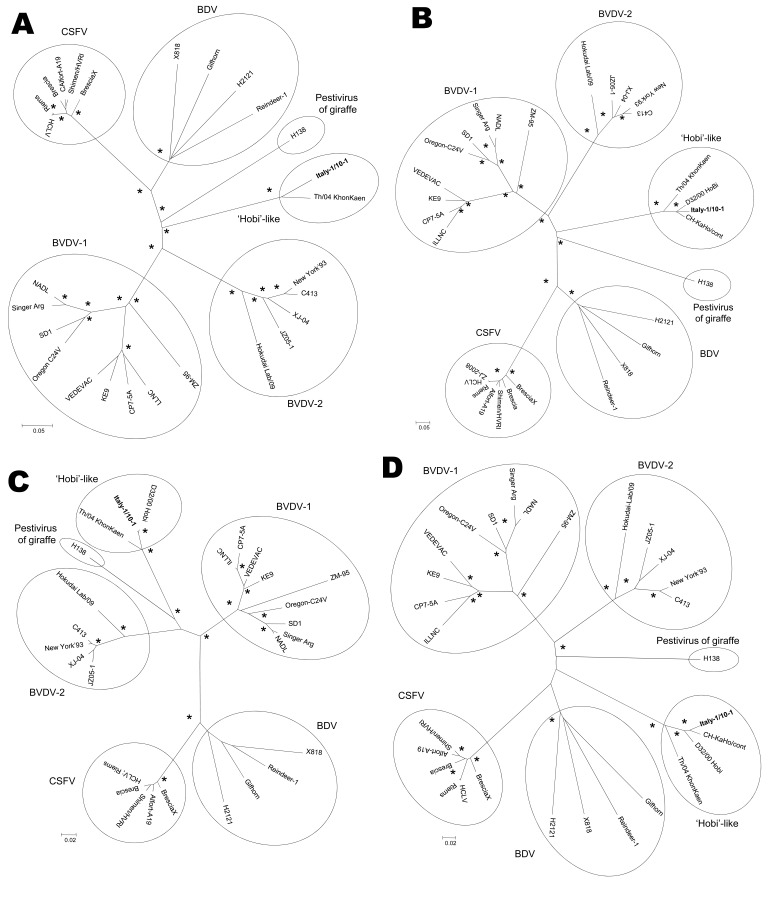
Neighbor-joining unrooted trees based on the full-length genome (A), E2 (B), 5′ untranslated region (C), and N^pro^ (D) sequences of members of the genus *Pestivirus*. For phylogenetic tree construction, pestivirus sequences listed in the Table were used. Asterisks indicate strong statistical support for a node by a bootstrap value of 75%–100%. Scale bars represent estimated numbers of nucleotide substitutions per site. CSFV, classical swine fever virus; BVDV-1, bovine viral diarrhea virus type 1; BVDV-2, bovine viral diarrhea virus type 2; BDV, border disease virus.

We evaluated the pathogenic potential of strain Italy-1/10–1 in 2 seronegative 6-month-old calves; an additional calf served as control. The 2 challenged calves showed only mild clinical signs consisting of mucoserous nasal discharge, hyperthermia, and moderate leukopenia. The successful infection was confirmed by viremia and viral shedding through the nasal and fecal routes (data not shown). Seroconversion was demonstrated by using the BVDV-Ab SVANOVIR ELISA (Svanova Biotech AB, Uppsala, Sweden) and virus neutralization (*5*), with mean optical density value and virus neutralizing titer of 0.398 (cutoff 0.200) and 512, respectively.

## Conclusions

Traditionally, 4 different species have belonged to the genus *Pestivirus*, but in the past few years, new putative members have been described worldwide. In 2004, an atypical pestivirus, D32/00_HoBi, distantly related to BVDV-1 and BVDV-2 was isolated from a batch of fetal calf serum collected in Brazil (*3*). Strictly related viruses were later identified in the blood of a buffalo in Brazil and in contaminated cell cultures in South America (*4*).

More recently, another atypical pestivirus, Th/04_KhonKaen, was isolated from a bovine serum sample during an epidemiologic survey in Thailand (*5*). Based on the genetic and antigenic divergence from known BVDV-1 and BVDV-2 strains, all these viruses were proposed as members of a new species of the genus *Pestivirus* (*5**,**8**,**10*).

The results of our study show that atypical pestiviruses also are circulating on the European continent. Infection caused by this new virus was associated with overt disease in cattle. Although experimental infection of seronegative calves induced only mild disease, Koch’s postulates were fullfilled, as demonstrated by some clinical signs, viremia, viral shedding, and seroconversion.

At the genetic level, the HoBi-like strain from Italy was more strictly related to the viruses from Brazil than to the isolate from Thailand. Considering that the virus has been repeatedly detected in batches of fetal calf serum (*3**,**11*), a possible introduction of the virus to the European continent through vaccines or other products prepared with contaminated bovine serum should be taken into account. Accordingly, previous reports suggest that BVDV may spread (and cause severe disease) through vaccination with contaminated products (*12**–**14*). By phylogenetic analysis of the full-length genome and E2 and N^pro^ regions, atypical pestiviruses formed a monophyletic cluster that was approximately equidistant from BVDV-1/BVDV-2 and BDV/CSFV, whereas the dendrogram obtained from 5′ UTR showed a closer relatedness to BVDVs. Thus, phylogeny may not support naming HoBi-like strains as BVDV-3 because the proposed nomenclature (*8**,**10*) does not reflect the genetic relationship among different pestivirus species.

Antigenic differences have been found between HoBi-like strains and BVDV-1/BVDV-2 through cross-neutralization assays (*3**,**5*). This difference also was evident in this study in that experimentally infected calves displayed high antibody titers by virus neutralization with the homologous virus, whereas only titers slightly above the cutoff were obtained by a commercial BVDV-1–based ELISA. The genetic and antigenic differences between HoBi-like strains and BVDV-1/BVDV-2 pose intriguing questions about the efficacy of commercially available BVDV vaccines and the need to develop specific vaccines against this new virus. In addition, BVDV surveillance programs may be affected by the poor sensitivity of commonly used PCRs with respect to atypical pestiviruses because of their genetic distance from BVDVs. Continuous epidemiologic surveillance will help assess the extent to which HoBi-like pestivirus strains are widespread in cattle populations worldwide and their impact on animal health and production, thus requiring specific immunization plans.
